# Trends in incidence and mortality risk for acromegaly in Norway: a cohort study

**DOI:** 10.1007/s12020-022-03275-6

**Published:** 2022-12-16

**Authors:** Camilla M. Falch, Nicoleta C. Olarescu, Jens Bollerslev, Olaf M. Dekkers, Ansgar Heck

**Affiliations:** 1 Section of Specialized Endocrinology, Department of Endocrinology, Oslo University Hospital, Oslo, Norway; 2 Faculty of Medicine, University of Oslo, Oslo, Norway; 3 Department of Clinical Epidemiology, Leiden University Medical Centre, Leiden, The Netherlands; 4 Department of Clinical Epidemiology, Aarhus University Hospital, Aarhus, Denmark

**Keywords:** Acromegaly, Epidemiology, Mortality, Cancer, Incidence, Prevalence

## Abstract

**Purpose:**

Recent data have shown a decreasing overall mortality in acromegaly over the last decades. However, cancer incidence and cancer-related mortality still appear to be increased. Our aim was to obtain updated epidemiological data from Norway in a clinically well-defined cohort with complete register-based follow-up.

**Methods:**

Patients diagnosed with acromegaly from South-Eastern Norway between 1999–2019 (*n* = 262) and age and sex matched population controls (1:100) were included (*n* = 26,200). Mortality and cancer data were obtained from the Norwegian Cause of Death and Cancer Registry. Mortality and cancer incidence were compared by Kaplan–Meier analyses and Cox regression; we report hazard ratios (HRs) with 95% confidence intervals (95% CI).

**Results:**

Median age at diagnosis was 48.0 years (interquartile range (IQR): 37.6–58.0). Mean annual acromegaly incidence rate was 4.7 (95% CI 4.2–5.3) cases/10^6^ person-years, and the point prevalence (2019) was 83 (95% CI 72.6–93.5) cases/10^6^ persons. Overall mortality was not increased in acromegaly, HR 0.8 (95% CI 0.5–1.4), cancer-specific and cardiovascular-specific mortality was also not increased (HR: 0.7 (95% CI 0.3–1.8) and 0.8 (95% CI: 0.3–2.5) respectively). The HR for all cancers was 1.45 (1.0–2.1; *p* = 0.052).

**Conclusion:**

In this large cohort study, covering the period 1999–2019, patients were treated with individualized multimodal management. Mortality was not increased compared to the general population and comparable with recent registry studies from the Nordic countries and Europe. Overall cancer risk was slightly, but not significantly increased in the patients.

## Introduction

Acromegaly is a chronic disease caused by excessive secretion of growth hormone (GH) most often from a somatotroph pituitary adenoma, with subsequently increased levels of insulin-like growth factor 1 (IGF-1) [1]. Based on recent studies, the estimated prevalence is 28–137 cases/million inhabitants and the incidence varies between 2–11 cases/million per year [2–5], hence acromegaly is considered an orphan disease as defined by the European Union (European Medicines Agency, 02.09.2019). The clinical manifestations of acromegaly are caused by systemic effects related to the prolonged exposure to GH/IGF-1 excess (musculoskeletal, cardiovascular and metabolic comorbidities) and the local tumor extension (visual-field defects, cranial-nerve palsy and hypopituitarism) [1]. The metabolic complications, including insulin resistance and diabetes mellitus, increase the risk of cardiovascular-related morbidity and mortality [1, 6]. Further, increased levels of IGF-1 has been associated with increased risk for several malignancies [7], and studies have suggested GH and IGF-1 to facilitate a tumor microenvironment and neoplastic growth in the colon [8, 9].

Surgery is the only curative treatment. However, due to delayed diagnosis most patients with acromegaly present with macroadenomas (65–79%) and frequently invasive tumors [10]. Thus, the cure rate is disappointingly low when performed as primary treatment in clinical practice [11–15]. Treatment algorithms with focus on improving the surgical cure rate by preoperative medical treatment have been developed during the last two decades. The purpose of this individualized approach was to improve therapeutic outcomes and reduce the need for complicated and costly long-term medical therapy [10, 13, 15, 16]. The concept is based on relatively small prospective studies showing an improvement in surgical cure rate in newly diagnosed patients with acromegaly following somatostatin analogue (SSA) pretreatment, as compared to direct surgery [4, 17–19]. However, as the few RCT’s on the topic mostly provide short-time observation on the primary endpoint being cure rate, the concept is still debatable and well-planned studies with longer postoperative observation are warranted.

Before the millennium, mortality in patients with acromegaly was reported to be increased by two- to three- fold compared to the general population. However, since the millennium overall mortality rates have declined [2]. This change has been ascribed to the modern, multimodal therapy [2, 20]. According to a large meta-analysis, malignancies have become the leading cause of death [2]. Recent studies suggest that the type of cancers related to mortality in acromegaly are diverse, and not restricted to those traditionally associated with acromegaly, such as colorectal and thyroid cancer [2]. Thus, studies indicate that when mortality in acromegaly declines by modernized treatment, causes of death in acromegaly shift towards ageing and environmental factors, similar as in the general population [2].

Recent meta-analyses have shown an elevated risk of cancer in patients with acromegaly [21]. However, conflicting results regarding cancer risk in patients with acromegaly have been described, including population based studies from the United Kingdom and Germany that showed a lower cancer incidence in patients when compared to controls (standardized incidence ratio (SIR) 0.76 (95% confidence interval (CI) 0.60–0.95) and 0.75 (95% CI: 0.55–1.00), respectively) [22, 23].

The aim of the present single center cohort study was to investigate incidence, prevalence, overall mortality and the risk of cancer in a clinically well-defined cohort of patients with acromegaly with complete register-based follow-up.

## Material and methods

### Study design and population

Oslo University Hospital (OUS) is the tertiary referral center for patients with acromegaly in the South-Eastern Health Region of Norway, which is the regional health authority for about 3 million inhabitants, approximately 56% of the total Norwegian population (South-Eastern Norway Regional Health Authority, 16.11.2020). Between August 1999 and December 2019, 262 patients with newly diagnosed acromegaly were managed at OUS (Section of Specialized Endocrinology). This comprises patients included in the Preoperative Treatment of Acromegaly (POTA) study between 1999 and 2005 [4, 17, 18], and thereafter patients from our internal pituitary quality registry. The included patients underwent a standardized diagnostic work up, as established by the POTA protocol [4, 17, 18]. The patients were followed prospectively, and clinical, biochemical and radiological findings were recorded during a standardized set of serial visits, from diagnosis at baseline and following the treatment on a yearly basis. Patients with invasive and/or macroadenomas were usually offered primary SSA treatment for an individualized time period (in general minimum 6 months), before subsequent transsphenoidal surgery. Treatment was carefully tailored by a multidisciplinary approach according to the most recent recommendations [10, 12–16]. Treatment information during the time of follow-up including surgical procedures, medical treatment (1^st^ generation SSAs, Pasireotide, dopamine agonists (DAs), GH receptor antagonists (GHRAs)) and radiotherapy was recorded consecutively during regular visits. During the study period, different assays for GH and IGF-1 were used, and we used morning GH levels (μg/L) for the statistical analysis as described previously [24]. IGF-1 is presented as the ratio of measured IGF-1 values, divided by the age-specific upper limit of normal (IGF-1/ULN).

A control cohort was obtained from the general population of the South-Eastern Health Region of Norway by the National Population Register and the Norwegian Tax Administration. The comparison cohort consisted of 100 age - and gender matched persons for every patient (*n* = 26,200). Date of diagnosis was considered the index date for the acromegaly patients and the matched controls. Demographics for the total population of the South-Eastern Health Region of Norway were obtained by The National Statistical Institute of Norway (National Statistical Institute of Norway, 04.11.2020).

Follow-up started at the date of diagnosis and the matched index date for the control cohort members. The follow-up period ended at the time of death or at the end of study (December 31, 2019).

### Cause of death and cancer data

We received data regarding cause and date of death, and cancer diagnosis and localization from the Norwegian Cause of Death Registry (Norwegian Cause of Death Registry, 03.05.2022) and Cancer Registry of Norway (Cancer Registry of Norway, 02.05.2022). The cancer diagnoses were coded according to the International Classification of Diseases 10^th^ Revision (ICD-10), and categorized into major cancer groups. Registry entries defined as benign by the Cancer registry of Norway (including pituitary adenomas) were excluded from the cancer analyses. International rules for multiple primary cancers (ICD-0 third edition) were used to define multiple primary neoplasms when reporting the data on cancer diagnoses and incidences [25]. For estimation of cancer incidence, only cancer diagnoses established after the index date were considered. However, all established cancer diagnoses between date of birth and end of follow-up were considered, when describing cancer events over time in relation to date of acromegaly diagnosis or the corresponding index date in the control cohort. Cancer events were adjusted for persons at risk per year.

### Statistical analyses

We estimated the annual incidence rate of acromegaly per 10^6^ persons-years based on the total population of South-Eastern Health Region of Norway for each calendar year, and the mean annual incidence rate. The point prevalence was estimated per million inhabitants in 2019, the last year of the study. Kaplan–Meier analysis was used to generate survival curves and Cox regression was used for time to event analysis. Using the comparison cohort as a reference, hazard ratios (HRs) with 95% CIs for mortality were estimated. Additionally, we divided the study period into three periods, and HRs for the periods 1999–2005, 2006–2012 and 2013–2019 were estimated, to analyze the potential change in mortality over time. To investigate cause-specific mortality, cause of death was categorized into main groups according to the leading causes of death observed in the acromegaly cohort; cardiovascular (any cardiovascular death), cancer (any cancer death) and other (any death that was not cardiovascular or cancer). Cox regression was used to investigate potential risk factors for mortality for predefined baseline characteristics (age, sex, tumor size, IGF-1/ULN values and first treatment modality). As only the first treatment was considered and this was close to baseline, immortal time bias was not considered an issue. We calculated and compared cancer incidence in the case and the control cohorts. Data are presented as median (interquartile range (IQR)) for continuous measures, and n (%) for categorical variables. For comparison of medians, Wilcoxon rank-sum test was used. Statistical analyses were executed by using STATA version 16.1.

## Results

### Patient characteristics

A total of 262 patients with acromegaly and 26,200 age- and sex-matched controls were included, 50.4% women. The mean follow-up time was 9.2 (SD: 6.0, range (0.0–20.4)) years. The median age at diagnosis was 48.0 years (37.6–58.0) and was constant over time. There was no major difference in median age at diagnosis between men and women (49.0 (37.8–59.2) and 47.7 (37.5–57.2), respectively (*p* = 0.700)). In the acromegaly cohort the median baseline GH and IGF-1/ULN were 7.9 (3.4–18.8) μg/L and 2.5 (1.8–3.4), respectively. At baseline, the median IGF-1/ULN was 2.4 (1.6–3.1) in women and 2.6 (1.9–3.5) in men. Radiological assessments were available for 229 patients, of whom 21% had microadenomas (<10 mm) and 79% had macroadenomas (≥10 mm). As a first treatment, 48% received surgery and 44% received 1^st^ generation SSAs (remaining listed in Table 1). Six percent was treated with radiotherapy at some time point, and 8% underwent multiple surgeries. At the last visit, median GH had decreased to 1.1 (0.4–2.8) μg/L and median IGF-1/ULN to 0.9 (0.7–1.1). Of a total of 240 patients with available IGF-1/ULN values and with more than at least one year follow-up time, 62% were in remission (defined as IGF-1/ULN levels < 1). Medical treatment for acromegaly (including 1^st^ generation SSAs, Pasireotide, DAs, GHRAs, or medical combination treatment) was received by 49% of patients, and 51% did not receive any treatment. Due to pituitary deficiency, 32% received hormone replacement therapy at the last visit (Table 1).

**Table 1:** Patients’ characteristics of the acromegaly cohort

	Total
	*N* = 262
Baseline	
Age at diagnosis	48.0 (37.6–58.0)
Women	47.7 (37.5–57.2)
Men	49.0 (37.8–59.2)
Sex (Women)	132 (50%)
Growth hormone (μg/L)^a^	7.9 (3.4–18.8)
IGF-1/ULN^b^	2.5 (1.8–3.4)
Women	2.4 (1.6–3.1)
Men	2.6 (1.9–3.5)
Tumor size (mm)^c^	15 (10–20)
Macroadenoma	180 (79%)
Microadenoma	49 (21%)

Treatment	
First treatment	
Surgery	125 (48%)
1st generation SSAs	114 (44%)
DAs	10 (4%)
Other^d^	11 (4%)
Radiotherapy^e^	16 (6%)
Multiple surgeries^f^	21 (8%)
Two	19 (7%)
Three	2 (1%)

Last visit^g^	
Growth hormone (μg/L)^h^	1.1 (0.4–2.8)
IGF-1/ULN^i^	0.9 (0.7–1.1)
Biochemical remission^j^	149 (62%)
Current therapy^k^	
1st generation SSAs	92 (36%)
Other^l^	32 (13%)
None	131 (51%)
Substitution therapy^m^	
No	163 (68%)
Yes	78 (32%)

Baseline is the visit at the department when acromegaly diagnosis was established. Last visit is the last visit at the department before end of study

*IGF-1/ULN* Insulin-like growth factor 1 upper limit of normal, *SSAs* somatostatin analogs, *DAs* dopamine agonists

^a^*N* = 164

^b^*N* = 206

^c^*N* = 229

^d^*N* = 260 and includes growth hormone receptor antagonists, medical combination treatment, no treatment and censored (due to lack of follow-up)

^e^*N* = 262

^f^*N* = 256

^g^*N* = 248, patients with follow-up time < 1 year were excluded.

^h^*N* = 207.

^i^*N* = 240.

^j^*N* = 240 and biochemical remission was defined by IGF-1/ULN levels < 1.

^k^*N* = 241.

^l^Includes DAs, growth hormone receptor antagonists, Pasireotide and medical combination treatment.

^m^*N* = 241 and includes patients that received hormonal therapy due to pituitary insufficiency.

### Incidence and prevalence

The point prevalence of acromegaly was 83 (95% CI: 72.6–93.5) cases/10^6^ in 2019. The mean annual incidence rate was 4.7 (95% CI: 4.2–5.3) cases/10^6^ persons, and remained constant over time.

### Mortality

**Fig. 1:**
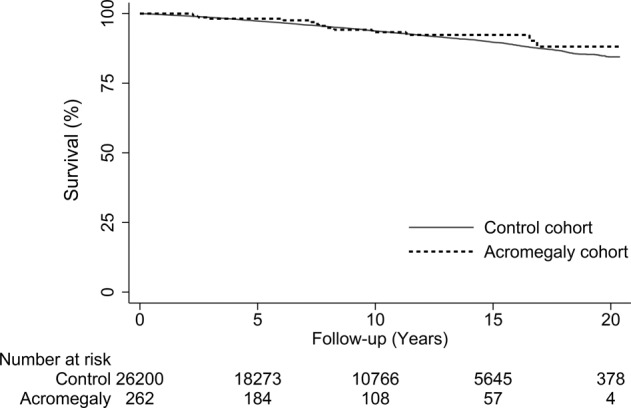
Mortality. Kaplan–Meier analysis of observed mortality rates among patients with acromegaly (dashed line) and matched controls (solid line), including number at risk for patients with acromegaly and controls for the study period

During the follow-up period, 14 patients (5%) with acromegaly died; 5 from cancer, 3 from cardiovascular disease and 6 from other causes (including multiple sclerosis, unspecified ileus, unspecified diabetes mellitus with renal complications, unexplained instantaneous death and missing). The overall mortality risk in acromegaly was not increased compared to the general population: HR 0.83 (95% CI: 0.49–1.41), *p* = 0.501 (Fig. 1).

**Fig. 2:**
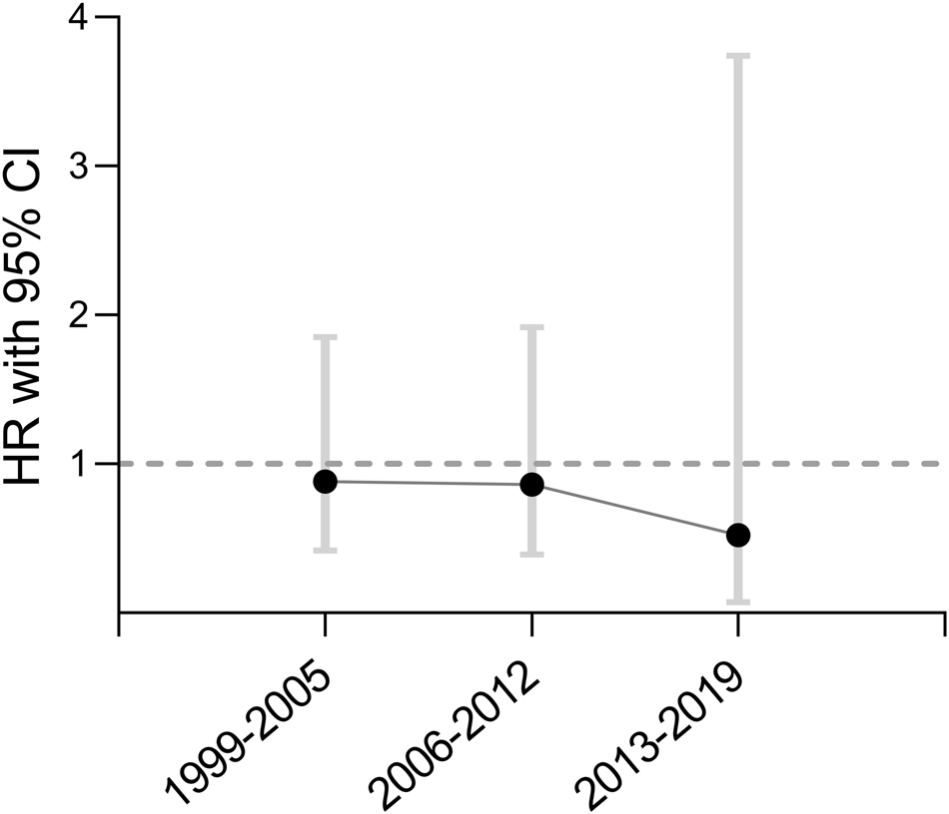
Mortality over three time periods. HRs (95% CIs) of mortality for patients diagnosed with acromegaly within time periods 1999–2005, 2006–2012 and 2013–2019

For patients diagnosed between 1999–2005, 2006–2012 and 2013–2019 the HRs for death were 0.88 (95% CI: 0.42–1.85), 0.86 (95% CI: 0.39–1.92) and 0.52 (95% CI: 0.07–3.74), respectively (Fig. 2).

Cancer-specific and cardiovascular-specific mortality risks were not clearly increased in patients with acromegaly (HR 0.74 (95% CI: 0.31–1.79) and 0.80 (95% CI: 0.26–2.48), respectively). Age was the only factor at baseline associated with mortality risk in acromegaly (HR 1.16 (95% CI 1.07–1.26)), whereas sex, IGF-1/ULN levels, tumor size at diagnosis, and first treatment modality (surgery or SSAs) did not influence mortality risk (Table 2).

**Table 2:** Baseline characteristics and mortality risk in patients with acromegaly

	Dead *N* = 14	Alive *N* = 248	HR	95% CI	*P*-value
Age at diagnosis	67.8 (59.2–76.8)	47.1 (37.4–56.7)	1.16	1.07–1.29	<0.001
Sex (Women)	10 (71%)	122 (49%)	2.21	0.44–11.15	0.336
IGF-1/ULN	2.5 (1.7–3.0)	2.5 (1.8–3.4)	0.64	0.32–1.28	0.208
Tumor size (mm)	17 (15–23)	14 (10–20)	1.04	0.98–1.10	0.169
First treatment (surgery)	5 (38%)	120 (50%)	0.92	0.17–5.07	0.921

Baseline characteristics of the patients that died and the patients alive during the study period. Risk of mortality is given in HRs, 95% CI and *p*-values in patients that died during the study period compared to alive patients (Cox regression).

*IGF-1/ULN* Insulin-like growth factor I upper limit of normal.

### Cancer

**Fig. 3:**
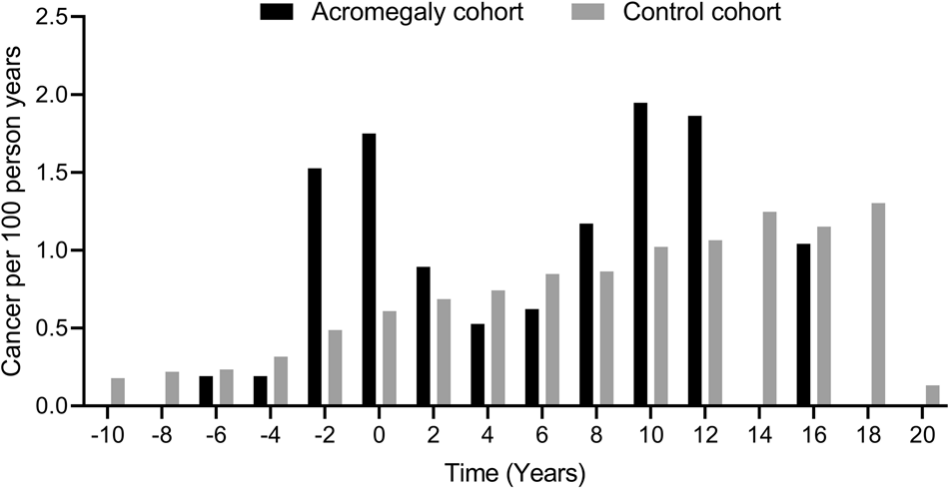
Cancer incidence rate over time in patients with acromegaly and matched controls. Cancer diagnoses are presented per 100 person years in the control and acromegaly cohort distributed in relation to time of acromegaly diagnosis/index date (Year 0). The time categories cover two years each, e.g., “0” covers the time interval from time of diagnosis/index date until two years after diagnosis, and “−2” covers two years before diagnosis until time diagnosis/index date

Of the patients with acromegaly, 28 (10.7%) developed cancer following the diagnosis of acromegaly, as compared to 2063 (7.9%) of the matched controls after the corresponding index date. The HR for all cancers was 1.45 (95% CI: 1.0–2.1), *p* = 0.052. There were 3 cases of thyroid cancer (1.1%) in the acromegaly cohort and 19 (0.1%) in the control cohort. The HR for thyroid cancers was increased in patients with acromegaly as compared to the controls (HR: 17.0 (95% CI: 5.0–58.1), *p* < 0.001). We found no increased risk for other cancers, however, there was a borderline significant increased risk of prostate cancer in male patients with acromegaly (HR 2.05 (95% CI: 0.97–3.37), *p* = 0.060) (Table 3). Figure 3 illustrates all established cancer diagnoses in the study adjusted for persons at risk per year. There was a total of 48 cancer diagnoses in the acromegaly cohort, and 3211 in the control cohort, when including both cancers diagnosed before and after the diagnosis of acromegaly and index date, respectively. There was an increased rate of cancer diagnoses in the period around acromegaly diagnosis (±2 years) (Fig. 3).

**Table 3:** Cancer risk in patients with acromegaly and matched controls

Cancer	Incidences after study inclusion				
	Acromegaly	Control			
	cohort	cohort			
	*N* = 262	*N* = 26200	HR	95% CI	*P*-value
Overall	28 (10.7%)	2063 (7.9%)	1.45	1.00–2.10	0.052
Localized	11 (4.2%)	776 (3.0%)	1.52	0.84–2.76	0.168
Non-localized	12 (4.6%)	845 (3.2%)	1.50	0.85–2.66	0.162
Missing	5 (1.9%)	442 (1.7%)			
Breast	1 (0.4%)	259 (1.0%)	0.40	0.06–2.86	0.362
Prostate	7 (2.7%)	356 (1.4%)	2.05	0.97–4.37	0.060
Colorectal	5 (1.9%)	265 (1.0%)	2.05	0.84–4.97	0.113
Thyroid	3 (1.1%)	19 (0.1%)	16.98	4.96–58.10	<0.001
Kidney	1 (0.4%)	53 (0.2%)	1.86	0.26–13.51	0.537
Hematological	3 (1.1%)	171 (0.7%)	1.87	0.59–5.85	0.285
Lung	2 (0.8%)	202 (0.8%)	1.07	0.27–4.33	0.920
Other	6 (2.3%)	738 (2.8%)	0.88	0.39–1.97	0.755

Cancer incidences in patients with acromegaly and matched controls. Risk of cancer is given in HRs, 95% CI and p-values in patients compared to controls, and estimated for cancer incidences after study inclusion (Cox regression).

## Discussion

In the present prospective, single center cohort study from the South-Eastern Region of Norway, we found a constant incidence rate of acromegaly over time, and the mortality for patients diagnosed in the last two decades was persistently not elevated. There was a trend towards increased cancer incidence in patients with acromegaly. No acromegaly-related risk factors for death could be identified.

The prevalence and incidence of acromegaly in the present study are in line with previous estimates from the Nordic countries [4, 5, 26–29], indicating a good coverage of the patient population.

We did not demonstrate increased mortality in patients with acromegaly compared to the general population, in contrast to the slightly elevated rates in Sweden and Denmark [3, 26, 30, 31]. Possible explanations may be that the present analyses are based on a more recent cohort (1999–2019) than the analyses from Sweden (1987–2013 [3, 31] and 1991–2011 [30]) and Denmark (1991–2010 [26]). This is supported by the decline in mortality in Sweden in the patients diagnosed more recently [31], and is coinciding with the broader availability of effective medication for acromegaly and improved surgical and radiation techniques, enabling multi-modal, individualized treatment to patients with acromegaly. Although the overall mortality in the most recent Swedish publication was increased, the mortality in patients with biochemical control was not elevated, in contrast to non-controlled patients [30]. Due to few events, no firm conclusion on the most recently diagnosed patients could be drawn, as indicated by a broad CI in our study. However, similar trends with improved survival have been demonstrated previously in meta-analyses [2, 20].

In the present study, we could not identify any baseline characteristics with significant influence on mortality, except for age, as expected. In order to avoid immortal time bias, only baseline characteristics were included in our analysis [32]. The majority of our patients had pituitary macroadenomas, and almost half of them received SSAs as a primary treatment, because the probability for surgical cure was considered to be low in many of these cases. This practice was regularly implemented in the patients diagnosed in the early 2000s, when the first randomized study on preoperative SSA treatment was initiated [4]. This change in treatment, together with the modernized and individualized approach over the last twenty years, had an important effect on mortality. When mortality in acromegaly decreases towards the background population, causes of patient death shift towards causes in the general population [2]. Thus, the leading cause of death in the Norwegian population at present is malignancies, with cardiovascular disease as the second, in accordance with our findings in the patients with acromegaly (Norwegian Cause of Death Registry, 10.06.2021).

We observed an equal gender distribution and median age at diagnosis, in contrast to a meta-analysis demonstrating a moderate female predominance (53%), and a higher age at diagnosis in women as compared to men [33]. In comparison, a recent review found that acromegaly was more prevalent in women than in men, and women were older at diagnosis [34]. However, in the Nordics, the gender distribution was equal, and age at diagnosis for men and women was similar [26–28, 33, 34]. Studies derived from national registries, like the Danish and Swedish studies and the present study, are representative for a large, unselected populations, and are thus little prone to selection bias.

Cancer incidence increased with age in the control cohort as expected, whereas, in the acromegaly cohort cancer incidence peaked markedly around the time of acromegaly (Fig. 3). This could be ascribed to surveillance bias occurring after a cancer diagnosis resulting in the detection of acromegaly, or vice versa: Newly diagnosed acromegaly may prompt cancer screening or suspicion. In order to reduce surveillance bias when estimating cancer incidence and risk, we excluded cases of malignancy established before the diagnosis of acromegaly, in accordance with the recent Swedish population-based study [35]. In contrast to the registry-based cohort study from Denmark [21], we did not exclude cancers established one year after the acromegaly diagnosis in order to avoid discharging cancers that could be associated with acromegaly, and possibly reflect biologic effects of GH excess in the years before delayed acromegaly diagnosis. Similarly to our results, the referred studies from Scandinavia found an overall elevated cancer risk in patients with acromegaly, and the meta-analysis in the Danish publication added further support to these findings [21, 35]. Accordingly, a nationwide cohort study from Italy demonstrated an overall increased cancer risk in patients compared to the general population [36]. However, this is in contrast with the population based studies from the United Kingdom and Germany, where no increased cancer incidence in patients with acromegaly compared to the general population were demonstrated [22, 23].

Of all five thyroid cancer cases in our patient cohort, two were established before the diagnosis of acromegaly and three after. Only two of these five cases where diagnosed close to the diagnosis (+/− 2 years) of acromegaly. However, we cannot exclude that the elevated thyroid cancer risk in our study can be related to surveillance bias. As described, data on thyroid cancer in patients with acromegaly are controversial and the absolute numbers are low [21, 35, 36]. Interestingly, we found a borderline significant increased risk of prostate cancer in men with acromegaly. These findings are similar to the above mentioned Danish meta-analysis [21], but in contrast to the studies from the United Kingdom and Germany [22, 23]. Previous studies have shown that increased IGF-1 levels were associated with increased risk of prostate cancer [7]. For cancer categories with low incidence, no finite conclusions can be drawn, as there is a considerable risk for type 2 error.

The strengths of this study is that it is a single center study with patients followed according to a standardized management course and long follow-up interval for of up to 20 years, in combination with data from national health-registries, and the comparison with a large matched cohort based on the general population. Despite the single center design, the study cohort is population based representing unselected cases from over 3 million inhabitants. Although the study covers a large geographical area and population followed up to two decades, the absolute numbers of death and cancers were low.

## Conclusion

The incidence, prevalence, gender distribution and age at diagnosis of acromegaly in South-Eastern Norway is similar to data from the other Nordic countries. The mortality rates in the patient cohort, that has received modern multimodal therapies and active surveillance for acromegaly-related complications, was not different from the background population. As in other recent European studies, we found a trend towards an increased overall cancer risk. Cardiovascular- and cancer-related mortality were not different as compared to the general population.
